# TMEM9 activates Rab9-dependent alternative autophagy through interaction with Beclin1

**DOI:** 10.1007/s00018-024-05366-1

**Published:** 2024-07-30

**Authors:** Sohyeon Baek, Jae-Woong Chang, Seung-Min Yoo, JeongRim Choo, Sunmin Jung, Jihoon Nah, Yong-Keun Jung

**Affiliations:** 1 Department of Biological Sciences and Biotechnology, Chungbuk National University, Cheongju, 28644 South Korea; 2 School of Biological Sciences, Seoul National University, Seoul, 08826 South Korea; 3 Department of Pediatrics, University of Minnesota, Minneapolis, MN 55455 USA; 4 Department of Biochemistry, Chungbuk National University, Cheongju, 28644 South Korea

**Keywords:** Alternative autophagy, TMEM9, Beclin1, Rab9

## Abstract

Transmembrane protein 9 (TMEM9) is a transmembrane protein that regulates lysosomal acidification by interacting with the v-type ATPase complex. However, the role of TMEM9 in the lysosome-dependent autophagy machinery has yet to be identified. In this study, we demonstrate that the lysosomal protein TMEM9, which is involved in vesicle acidification, regulates Rab9-dependent alternative autophagy through its interaction with Beclin1. The cytosolic domain of TMEM9 interacts with Beclin1 via its Bcl-2-binding domain. This interaction between TMEM9 and Beclin1 dissociates Bcl-2, an autophagy-inhibiting partner, from Beclin1, thereby activating LC3-independent and Rab9-dependent alternative autophagy. Late endosomal and lysosomal TMEM9 apparently colocalizes with Rab9 but not with LC3. Furthermore, we show that multiple glycosylation of TMEM9, essential for lysosomal localization, is essential for its interaction with Beclin1 and the activation of Rab9-dependent alternative autophagy. These findings reveal that TMEM9 recruits and activates the Beclin1 complex at the site of Rab9-dependent autophagosome to induce alternative autophagy.

**Supplementary Information:**

The online version contains supplementary material available at 10.1007/s00018-024-05366-1.

## Introduction

Autophagy, a lysosome-dependent cellular catabolic process, encompasses LC3-dependent conventional autophagy and Rab9-dependent alternative autophagy, each characterized by distinct mechanisms [1, 2]. Conventional autophagy begins with phagophore formation, nucleated by the ULK1/2 complex and ATG proteins, leading to the conjugation of LC3 to phosphatidylethanolamine (LC3-II). Membranes are sourced from the ER, Golgi apparatus, mitochondria, and plasma membrane [2, 3]. The mature autophagosome fuses with lysosomes to degrade the encapsulated material. This canonical process is governed by core regulators such as Atg5, Atg7, and LC3, with over 35 autophagy-related genes [1, 3]. In contrast, Rab9-dependent autophagy operates without canonical core ATG proteins, such as Atg5, Atg7, Atg12, and LC3, relying on Rab9, a small GTPase, with membranes primarily derived from the trans-Golgi network and late endosomes [2, 4]. Electron microscopy analysis under Atg5-deficient conditions has revealed this non-canonical alternative autophagy pathway, where autophagosome/autolysosome generation and degradation occur independently of the Atg5-Atg7-LC3 dependent ubiquitin-like pathways [3, 4].

Ulk1, an essential initiator in both conventional and alternative autophagy, operates differentially in each pathway. While Ulk1 and Ulk2 redundantly participate in initiating conventional autophagy, Ulk1 stands as an indispensable initiator in alternative autophagy [5]. Following Ulk1 complex activation, autophagic vesicle nucleation commences via activation of the class III phosphatidylinositol 3-kinase (PtdIns3K) complex, including PtdIns3K and Beclin1, in both types of autophagy [6]. Nevertheless, the molecular mechanisms between conventional and alternative pathways governing the initiation, expansion, and closure of isolation membranes differ significantly. In conventional autophagy, ER membranes are commonly utilized as a membrane source for isolation membranes with the conjunction of LC3-II through the Atg5-Atg7-dependent ubiquitin-like processes [7, 8]. In contrast, alternative autophagy relies on the trans-Golgi network as the membrane source, aided by the Rab9 GTPase [4, 6]. However, the processes by which alternative autophagic membranes are formed in the absence of essential ubiquitin-like systems remain elusive.

Beclin1 is the first mammalian protein identified in the autophagy machinery. It functions as a molecular scaffold for autophagy signaling by interacting with various partners, thereby controlling autophagy activation, cellular trafficking, autophagosome-lysosome fusion, and even autophagy inhibition [9]. Beclin1 primarily forms a core complex by interacting with vacuolar protein sorting-associated protein 15 (VPS15) and VPS34 [9]. This core complex serves as a platform for multiple interactors involved in autophagy regulation, including Atg14 for autophagy induction, UVRAG for endosomal trafficking, and Rubicon for autophagy inhibition [10–12]. Furthermore, Beclin1 modulates both conventional and alternative autophagy pathways, suggesting its potential as a target for modulating these signaling pathways [4]. While many Beclin1 interactors involved in conventional autophagy have been identified, none specific to alternative autophagy have been reported.

TMEM9, a type I transmembrane protein, is predominantly localized in lysosomes, late endosomes, and multivesicular bodies. It consists of an N-terminal signal peptide, a single transmembrane spanning region, and three N-glycosylation sites in the N-terminal part of the mature form [13]. TMEM9 exhibits upregulation in colorectal cancer cells with hyperactivation of the Wnt/β-catenin signaling pathway, which is known to play a crucial role in intestinal tumorigenesis [14]. Moreover, TMEM9 is closely associated with inflammation, regulating TNFα-enhanced cytokine secretion through the canonical Wnt/β-catenin pathway [15]. The N-terminus of TMEM9 directly interacts with vacuolar-ATPase (v-ATPase) and its accessory protein ATP6AP2, facilitating vesicular acidification within the lysosome [14]. Despite being localized on the lysosomal membrane, the role of TMEM9 in autophagy remains unidentified.

This study aims to elucidate the molecular mechanism of alternative autophagy, finely regulated by the Beclin1 complex in the interaction with TMEM9. Specifically, our goal is to clarify the differential role of the Beclin1 complex in alternative autophagy compared to the conventional pathway, emphasizing its scaffolding activity.

## Materials and methods

### Reagents and plasmid constructs

The following chemicals used include bafilomycin A1 and tunicamycin (Sigma-Aldrich, St. Louis, MO, USA). Human TMEM9 cDNA was subcloned into pcDNA3-HA, pCMV-Flag, pCMV-RFP and, pEGFP. To construct serial deletion mutants of TMEM9, TMEM9 1-112 (TMEM9 ΔC), and TMEM9 90-183 (TMEM9 ΔN) were amplified by PCR and subcloned into pcDNA3-HA vector. Human Beclin1 cDNA was subcloned into pcDNA-HA, and pCMV-Flag. To construct serial deletion mutants of Beclin1, Beclin1 ΔBH3, Beclin1 ΔCCD, and Beclin1 ΔECD were amplified by PCR and subcloned into pCMV-Flag vector (pBeclin1 ΔBH3-Flag, pBeclin1 ΔCCD-Flag, and pBeclin1 ΔECD-Flag). All constructs were verified by DNA sequencing analysis. TMEM9 full-length was fused to the C-terminal Venus fragments of the pBiFC-VC vector and Beclin1 full-length, Beclin1 ΔBH3, Beclin1 ΔCCD, and Beclin1 ΔECD were fused to the N-terminal Venus fragments of pBiFC-VN vector. To construct TMEM9 shRNAs, forward and reverse 64-nucleotide fragments containing the 19-nucleotide human TMEM9 dsRNA hairpin coding sequence (5′-GAA GAT ATC CGG TGC AAA T-3′ corresponding to human TMEM9 169-187) as an inverted repeat separated by a 9-nucleotide-long hairpin region were annealed and inserted into the *Bgl*II/*Hin*dIII sites of pSUPER vector (Oligo Engine). To generate mutations of TMEM9, site-directed mutagenesis was conducted using wild-type TMEM9-flag and TMEM9-GFP vectors. The GFP-Rab9 and RFP-Rab9 were subcloned using the YFP-Rab9 vector that was previously described [16]. The GFP-LC3 and LAMP1-mCherry have been described previously [17].

### Antibodies and western blot analysis

The primary antibodies used include anti-LC3 (Novus Biologicals, NB600-1384), anti-Beclin1 (Cell signaling, 3495S), anti-Rab9 (Cell signaling, 5118S), anti-FLAG (Sigma Aldrich, F1804), anti-HA (Abcam, ab9110), anti-Bcl-2 (Sigma Aldrich, sc-5286), anti-αTubulin (Santa cruz technology, SC-5286). Anti-TMEM9 antibody was generated by following standard immunization procedures using the purified TMEM9 protein from E. coli. Western blot analysis was performed using standard techniques. Cells were lysed with sample buffer (10% glycerol, 2% SDS, 62.5 mM Tris–HCl, 2% β-mercaptoethanol, pH 6.8). Immunoprecipitation assays were performed as previously described [17]. Briefly, cells were lysed with modified RIPA buffer (10 mM Tris–HCl, 150 mM NaCl, 5 mM EDTA, 1 mM Na3VO4, 1% CHAPS). After pulldown with the appropriate antibodies, the same amounts of protein were separated by SDS-PAGE and transferred onto the polyvinylidene fluoride membrane (ATTO, AE-6667-P). Immunoblot analysis was then performed and visualized by the enhanced chemiluminescence method.

### BiFC assay

HeLa cells were transfected with VN or VC-fusion construct alone and Beclin1-Flag or in combination and incubated at 37 °C for 24 h. Cells were fixed using 4% PFA for 10 min and observed under confocal microscope.

### Cell culture and DNA transfection

HeLa and HEK293T cells were grown in Dulbecco's Modified Eagles Medium (DMEM) (Life Technologies Inc.) supplemented with 10% fetal bovine serum (FBS) at 37 °C. Cells were transfected for 24 h with the appropriate vector using Polyfect reagent (Qiagen) and selected with G418 (1 mg/ml) for 2 weeks to generate mixed populations (HeLa/shTMEM9-Mix).

### In vitro protein-binding assay

GST-fused proteins were immobilized by incubating with Glutathione Sepharose 4B and then incubated with [^35^S]-methionine-Beclin1 or Beclin1 (Promega) in a binding buffer [20 mm Tris–HCl (pH 7.2), 0.15 m NaCl, 0.2% Triton X-100 and protease inhibitors]. Beads were recovered and then washed with the binding buffer or lysis buffer A [50 mm Tris–HCl (pH 7.4), 300 mm NaCl, 1% Triton X-100, 0.1% BSA and protease inhibitors] several times and the bound proteins were subjected to SDS-PAGE.

### Confocal microscopy

HeLa cells were fixed with fresh 4% paraformaldehyde in PBS for 10 min at room temperature and washed with PBS. The sample was examined with an LSM-880 with Airyscan confocal imaging system (Carl Zeiss AG) using 63 × magnification and Zeiss LSM image browser software (Carl Zeiss).

### Immunostaining

Cells were fixed in 4% paraformaldehyde for 10 min and then permeabilized using 0.1% TritonX-100 in PBS. Cells were then blocked with 3% BSA in permeabilization buffer for 1 h at room temperature and stained with anti-Flag antibody for 2 h at room temperature. After washing, the cells were stained with Alexa Fluor™ Plus 405 for 1 h at room temperature and mounted in an antifade mounting medium.

### Assessment of autophagy

To assay conventional autophagy, the number of GFP-LC3-positive puncta per cell was determined by counting cells (n > 100 cells). LC3-II levels were assessed by western blotting using an anti-LC3 antibody (Novus Biologicals). LC3-II and αTubulin signals on western blots were quantified by densitometric analysis using ImageJ software. To assay alternative autophagy, the number and the size of the GFP-Rab9-positive puncta were analyzed using ImageJ software (n > 50 cells). Rab9 levels were assessed by western blotting with or without bafilomycin A1 treatment.

### Statistical analysis

Statistical analyses were performed using GraphPad prism. All data are expressed as mean ± SD. Results comparing three or more samples were analyzed using ANOVA and Bonferroni tests; comparisons between two samples were analyzed using Student’s t tests. Values of p < 0.05 were considered to be significant.

## Results

### The lysosome-resident protein TMEM9 interacts with the autophagy regulator Beclin1.

TMEM9 has recently been identified as a vesicular acidification regulator in the lysosomal membrane [14]. The ability of TMEM9 to control lysosomal pH is regulated by its interaction with v-ATPase. Given that v-ATPase-dependent lysosome acidification is a crucial cellular event in autophagosome maturation [18], we have investigated whether TMEM9 is involved in autophagy machinery. Subcellular localization of TMEM9 was examined using Rab5, Rab7, and LAMP1 as early endosome, late endosome, and lysosome marker proteins, respectively (Supplementary Fig. S1a–S1c). As previously reported [13], subcellular localization of TMEM9-RFP significantly overlapped with the lysosomal protein LAMP1 and late endosome protein Rab7 under basal and autophagy-inducing conditions. Additionally, it displayed slight colocalization with Rab5-positive early endosomes under control and autophagy-inducing conditions. (Supplementary Fig. S1a–S1d).

**Fig. 1:**
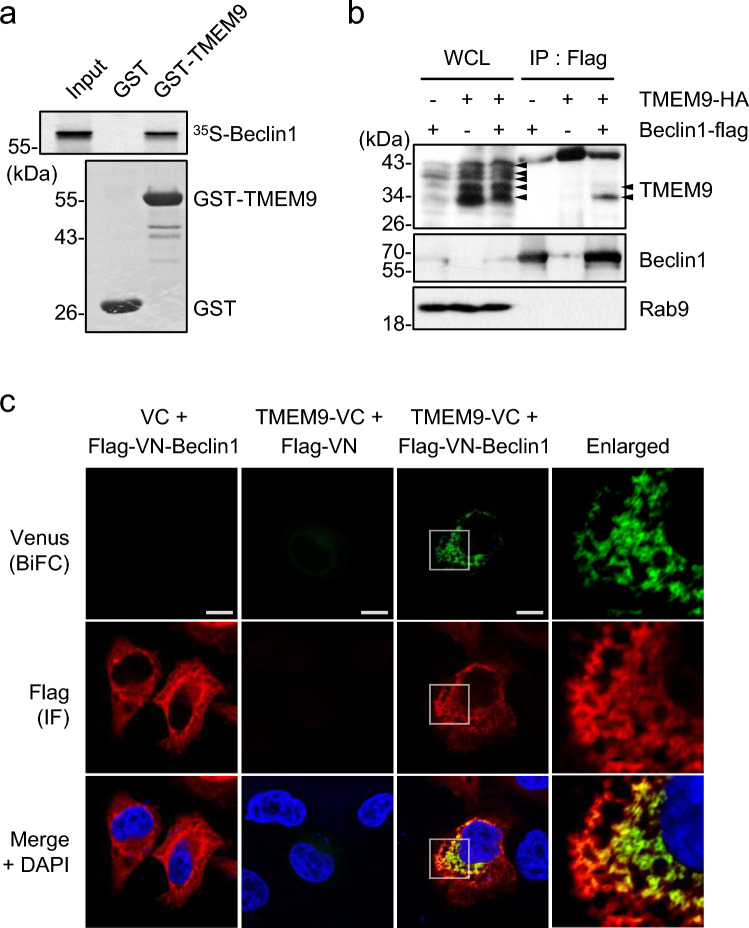
Lysosomal protein TMEM9 interacts with Beclin1. **a** GST and GST-TMEM9 proteins were purified by glutathione (GST)-agarose beads and incubated with the ^35^S-labeled in vitro translated Beclin1. After pulled-down with GST-agarose beads, the reaction products were separated by SDS-PAGE and autoradiography. Input indicates 10% of the in vitro translated Beclin1 protein. **b** HEK293T cells were transfected with TMEM9-HA and Beclin1-flag as indicated and subjected to immunoprecipitation (IP) assay using Flag-M2 magnetic beads. Whole-cell lysates (WCL) and the immunoprecipitates were analyzed by western blotting using antibodies against TMEM9, Beclin1, and Rab9. **c** HeLa cells were cotransfected with the VN, VC, TMEM9-VC, and VN-Beclin1 as indicated, and the BiFC assay was monitored under a confocal microscope. Scale bar: 10 μm

Among the various autophagy regulators, Beclin1 facilitates generating autophagosomes and regulating endocytosis and the autophagosome-lysosome fusion process through interaction with its interactomes [10, 19, 20]. Due to the close relationship between Beclin1 and lysosome, we initially investigated the molecular connection between Beclin1 and TMEM9 in the regulation of autophagy. An in vitro pull-down assay using purified GST-TMEM9 protein and ^35^S-Met-labeled Beclin1 demonstrated a direct interaction between TMEM9 and Beclin1 (Fig. 1a). To validate this interaction in the cellular system, TMEM9-HA and Beclin1-flag constructs were transfected into HEK293T cells. Western blotting revealed multiple slow-migrating signals of the overexpressed TMEM9 (Fig. 1b), which are glycosylated TMEM9 [13]. Immunoprecipitation (IP) assays confirmed the interaction between TMEM9 and Beclin1 in HEK293T cells (Fig. 1b). Additionally, a biomolecular fluorescence complementation (BiFC) assay, that is useful to visualize protein–protein interaction in live cells [21], was performed using TMEM9-fused C-terminal Venus (TMEM9-VC) and Beclin1-fused N-terminal Venus (VN-Beclin1). Compared to control cells that do not exhibit any BiFC signals, the intensity of the BiFC signals was apparently observed in HeLa cells expressing both TMEM9-VC and VN-Beclin1. Moreover, a significant area of the BiFC signal colocalized with Beclin1 (Fig. 1c). These results indicate that TMEM9 binds to Beclin1 in vitro and in cells.

### The cytosolic portion of TMEM9 binds to the BH3 domain of Beclin1

**Fig. 2:**
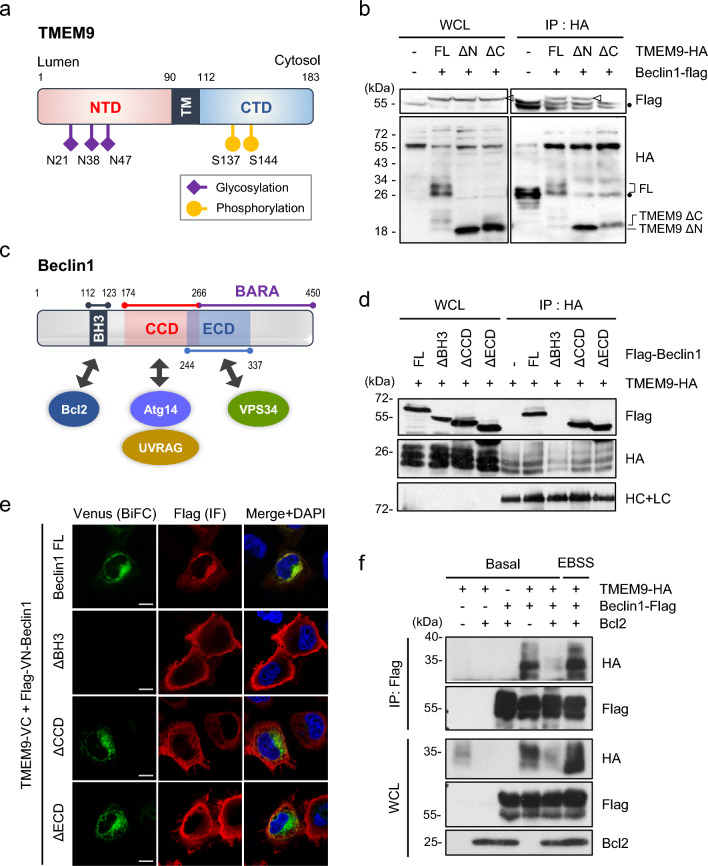
TMEM9 interacts with Beclin1 through its C terminus domain in a competitive manner with Bcl-2. **a** TMEM9 domains and the predicted sites of posttranslational modifications, including glycosylation and phosphorylation. NTD, N-terminal domain; CTD, C-terminal domain. **b** HEK293T cells were co-transfected with Beclin1-flag and either HA-tagged TMEM9 or its deletion mutants (ΔN; 90-183 residues, ΔC;1-90 residues) for 48 h. Cell lysates were immunoprecipitated (IP) using an anti-HA antibody and analyzed by western blotting using anti-flag and anti-HA antibodies. **c** Schematic representation of Beclin1 domains and its identified interacting partners. **d** HEK293T cells were co-transfected with TMEM9-HA and full-length (FL) or deletion mutants (∆) of Beclin1-flag. Cell lysates were immunoprecipitated (IP) as indicated in **b**. **e** HeLa cells were cotransfected with TMEM9-VC and VN-Beclin1 (FL) or its deletion mutations and visualized under a confocal microscope. Scale bar: 10 μm. **f** HEK293T cells were co-transfected with TMEM9-HA, Beclin1-flag, and Bcl-2 as indicated and incubated in basal medium or EBSS medium for 6 h. Cell lysates were immunoprecipitated (IP) using an anti-FLAG antibody and analyzed by western blotting using anti-flag, anti-HA, and anti-Bcl2 antibodies

To further delineate the interaction property between Beclin1 and TMEM9, we performed IP assays using Beclin1 and various deletion mutants of TMEM9, including the luminal part (TMEM9 1-90) and the cytosolic form (TMEM9 90-183) as described in the schematic image (Fig. 2a). The subcellular distribution of Beclin1 has been reported to localize in the cytoplasmic region or near the membrane, where are nucleated autophagosomes [22]. IP assays revealed that deletion of the C-terminal region, but not N-terminus, abolished their interaction (Fig. 2b), indicating that the C-terminal region of TMEM9 is essential for the interaction with Beclin1. We also examined the interacting domain of Beclin1 to TMEM9. The functions of Beclin1 are governed by the interaction with specific proteins that primarily determine its subcellular localizations (Fig. 2c) [23]. Examination of the interaction revealed that BH3 domain, crucial for interacting with Bcl-2, was necessary for its binding to TMEM9 (Fig. 2d). Again, we evaluated the interaction using the BiFC system and confirmed that Beclin1 lacking BH3 domain didn’t bind to TMEM9 (Fig. 2e). Collectively, these results suggest that the cytosolic domain of TMEM9 is able to interact with the BH3 domain of Beclin1.

Given that Beclin1 interacts with Bcl-2 through BH3 domain, and Bcl-2 dissociates from Beclin1 in response to autophagy-inducing conditions such as starvation [19], we investigated whether TMEM9 competes with Bcl-2 and promotes the dissociation of the Beclin1-Bcl-2 complex upon autophagy activation. With IP assays in HEK293T cells expressing Beclin1-flag, TMEM9-HA, and Bcl-2, ectopic expression of Bcl-2 reduced the interaction between Beclin1 and TMEM9 (Fig. 2f). Intriguingly, upon inducing autophagy with nutrient depletion (EBSS), the interaction between Beclin1 and TMEM9 was enhanced even in the presence of Bcl-2 (Fig. 2f). These findings indicate that TMEM9 competes with Bcl-2 to interact with Beclin1, and this interaction is altered by autophagy activity.

### The lysosomal localization of TMEM9 is essential for the interaction with Beclin1.

As N-linked glycosylation is crucial for membrane protein folding, stability and other cellular functions [24], glycosylation of lysosomal proteins like TMEM9 is required for the proper folding and trafficking to the lysosome. While the whole protein structure of TMEM9 has not been characterized, we predicted the expected structure and regions of post-translational modifications (PTMs) of TMEM9 using AlphaFold (Supplementary Fig. S2a). TMEM9 possesses three potential glycosylation asparagine residues (N21, N38, and N47) in the lysosomal luminal region and two putative phosphorylation serine residues (S137 and S144) in the cytosolic region. To assess the functional significance of TMEM9 PTM, various site-directed mutants of TMEM9 were generated (Supplementary Fig. S2b). Three glycosylated TMEM9 bands were affected by its point mutations or tunicamycin treatment as previously reported (Supplementary Fig. S2c and S2d) [13]. As Beclin1 binds to the C-terminus of TMEM9, we examined whether the phosphorylation status of TMEM9 affects its interaction with Beclin1. IP assays revealed single mutations (S137A, S137D, S144A, S144D) and dual mutations (2SA, 2SD) in TMEM9 for the phosphorylation-defective (S137A, S144A, S137A/S144A) or -mimic (S137D, S144D, S137D/S144D) mutants did not affect its binding to Beclin1 under basal and autophagy-inducing conditions (Supplementary Fig. S2e and S2f).

**Fig. 3:**
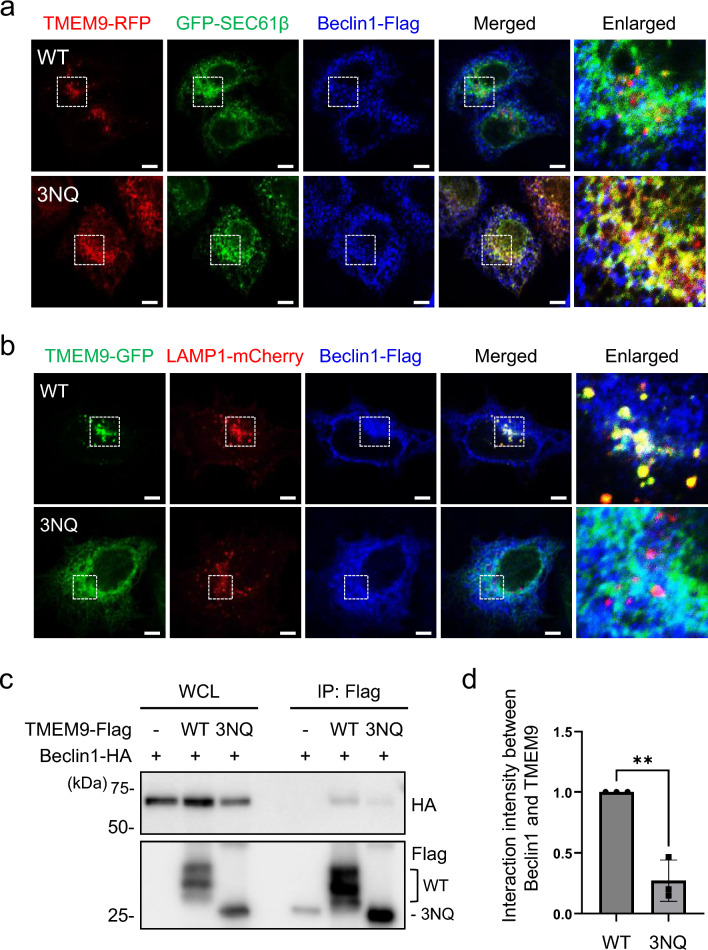
The glycosylation of TMEM9 is essential for its lysosomal localization and interaction with Beclin1. **a** and **b** HeLa cells were co-transfected with Beclin1-Flag, TMEM9-RFP (WT or 3NQ) (**a**) or TMEM9-GFP (WT or 3NQ) (**b**) and either GFP-SEC61β (**a**) or LAMP1-mCherry (**b**) for 12 h. Cells were immunostained with an anti-Flag antibody and observed under a confocal microscope. Scale Bar, 5 μm. **c** and **d** HEK293T cells were co-transfected with Beclin1-HA and either TMEM9-WT-flag or TMEM9-3NQ-flag. Cell lysates were immunoprecipitated (IP) using Flag-M2 magnetic beads and were analyzed by western blotting using anti-HA and anti-flag antibodies (**c**). The signals of TMEM9 and Beclin1 on the blots were quantified with ImageJ (**d**). The data were mean $\pm$ s.d. of three independent experiments. **p < 0.01

Next, we analyzed whether the N-linked glycosylation of TMEM9 is necessary for its interaction with Beclin1. Single mutations replacing asparagine 21, 38, or 47 with glutamine (N21Q, N38Q, N47Q) in TMEM9 did not affect the lysosomal localization of TMEM9 or its interaction with Beclin1 (Supplementary Fig. S2e and S2g). Interestingly, the triple mutation of all glycosylation sites to glutamine (3NQ; N21Q/N38Q/N47Q) yielded different consequences compared to single mutations. Confocal microscopy revealed that wild-type TMEM9 colocalized with LAMP1-positive lysosome together with Beclin1, whereas TMEM9 3NQ-GFP mutant was largely retained in the endoplasmic reticulum (ER) (Fig. 3a) and was found in neither the lysosome nor Beclin1 (Fig. 3b). Moreover, unlike wild-type TMEM9, we found that TMEM9-3NQ mutant was unable to interact with Beclin1 (Fig. 3c and d). These results suggest that the N-linked glycosylation of TMEM9 is essential for its lysosomal localization and interaction with Beclin1.

### TMEM9 is not involved in LC3-dependent conventional autophagy

**Fig. 4:**
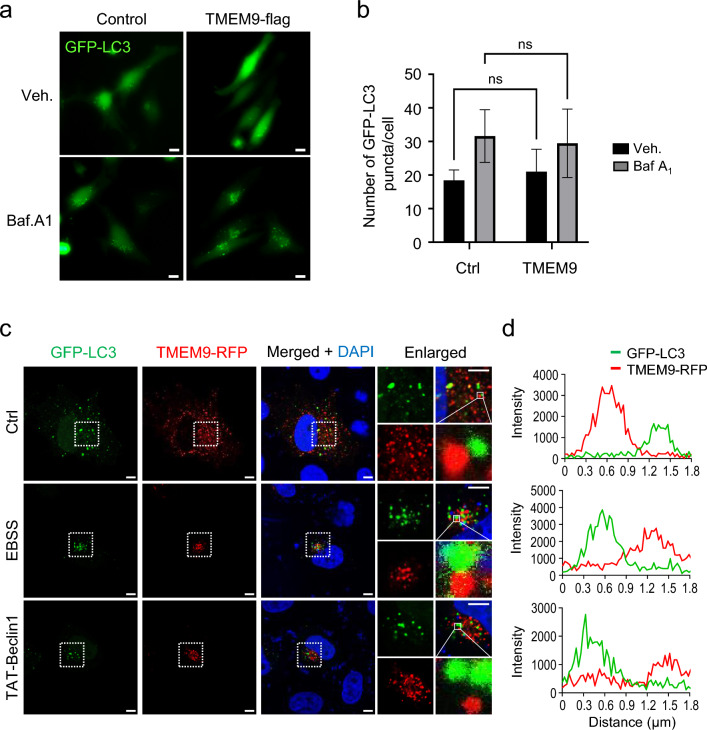
**Ectopic expression of TMEM9 does not induce LC-dependent conventional autophagy.** **a** and **b** HeLa cells were co-transfected with GFP-LC3 and TMEM9-flag for 24 h, treated with 20 nM bafilomycin A_1_ (Baf.A1) for 6 h, and then observed under the fluorescent microscope. Scale bar, 50 μm (**a**). The numbers of LC3 dots per cell on images in (**a**) were counted in (**b**). **c** and **d** HeLa cells were co-transfected with GFP-LC3 and TMEM9-RFP for 24 h, incubated with basal medium, EBSS medium, or 10 μM TAT-Beclin1 for 6 h, and observed under confocal microscope (**c**). Scale Bar, 5 μm. Fluorescent intensities of GFP (green) and RFP (red) on the indicated area in enlarged images of (**c**) were measured with ZEISS ZEN software (**d**)

**Fig. 5:**
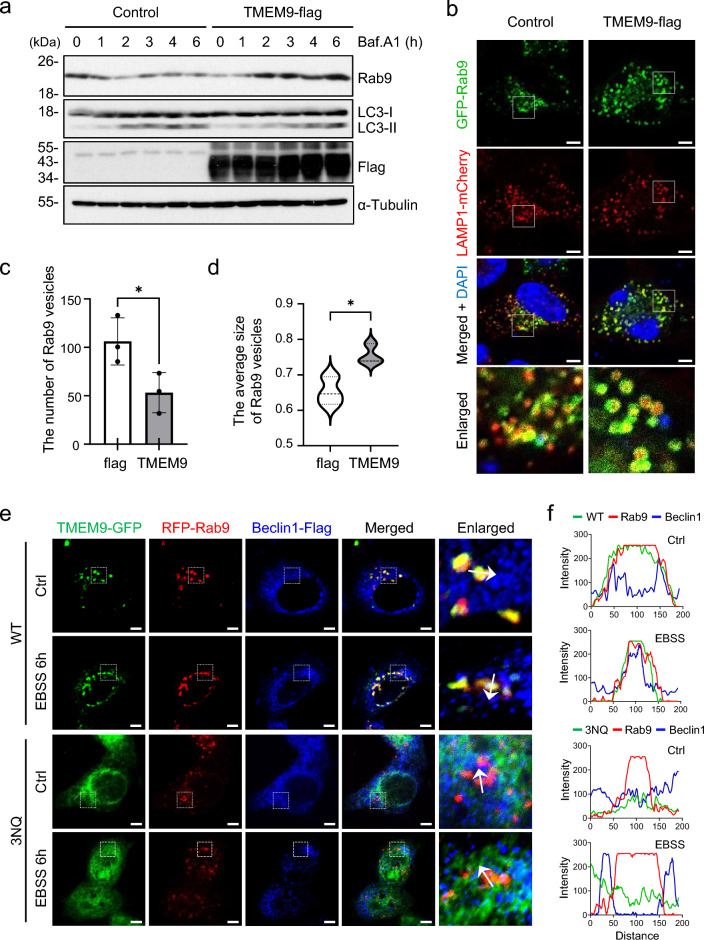
TMEM9 activates Rab9-dependent alternative autophagy. **a** HeLa cells were transfected with control or TMEM9-flag for 24 h, treated with 20 nM of bafilomycin A_1_ (Baf.A1) for the indicated times and analyzed with western blotting using anti-Rab9, anti-LC3, anti-flag, and anti-αTubulin antibodies. **b**–**d** HeLa cells were co-transfected with TMEM9-flag, GFP-Rab9, and LAMP1-mCherry in an indicated manner for 24 h and observed under the confocal microscope (**b**). The numbers (**c**) and the sizes (**d**) of Rab9-positive vesicles on the images were analyzed. The data were mean $\pm$ s.d. of three independent experiments. *p < 0.05. Scale Bar, 5 μm. **e** and **f** HeLa cells were co-transfected with RFP-Rab9, Beclin1-Flag, and either TMEM9-WT-GFP or TMEM9-3NQ-GFP for 27 h, incubated in control basal (Ctrl) or EBSS medium for 6 h. Cells were immunostained with and anti-Flag antibody and observed under a confocal microscope (**e**). Scale Bar, 5 μm. Fluorescent intensities of GFP (green), RFP (red), and Alexa Fluor™ Plus 405 (Blue) on the images are measured with ZEISS ZEN software (**f**)

To evaluate the role of TMEM9 in autophagy, we examined whether TMEM9 is involved in the Atg5-12 and LC3-dependent conventional autophagy pathway. Using GFP-LC3 as a general autophagy indicator [25], we analyzed effects of TMEM9 expression on GFP-LC3 puncta formation in HeLa cells. Unexpectedly, neither knockdown nor overexpression of TMEM9 significantly altered the numbers of GFP-LC3-positive puncta (Fig. 4a, b and Supplementary Fig. S3). In addition, western blot analysis revealed that overexpression of TMEM9 did not affect LC3-I conversion into LC3-II, a common marker of conventional autophagy in the presence or absence of Bafilomycin A1, a lysosome inhibitor (Fig. 5a). To further assess whether TMEM9 affects the fusion of autophagosomes with lysosomes during autophagic flux, we examined colocalization between TMEM9-RFP and GFP-LC3 under basal and autophagy-inducing conditions. Although GFP-LC3 dots increased under starvation and treatment with the autophagy-inducing peptide TAT-Beclin1 [26], TMEM9-positive vesicles did not overlap with the LC3-dependent autophagic vesicles under both basal and autophagy-inducing conditions (Fig. 4c). Collectively, these results suggest that TMEM9 is not involved in LC3-dependent conventional autophagy activation.

### TMEM9 regulates Rab9-dependent alternative autophagy

Autophagy is also regulated by LC3-independent and Rab9-dependent alternative pathways and Rab9-dependent alternative autophagy is governed by Beclin1 [4]. Thus, we further addressed the role of TMEM9 in alternative autophagy. With western blot analysis, we observed that compared to control cells, the level of Rab9, a marker of alternative autophagy, gradually increased by TMEM9 overexpression in HeLa cells upon Bafilomycin A1 treatment (Fig. 5a). On the other hand, LC3-II conversion was not affected under the same condition. Further, we assessed effects of TMEM9 expression on the Rab9-positive vesicle formation by co-transfecting HeLa cells with TMEM9-flag, GFP-Rab9, and LAMP1-mCherry. Interestingly, TMEM9 overexpression resulted in the enlargement of Rab9-positive vesicles rather than an increase in their numbers (Fig. 5b–d). Particularly, the average vesicle diameter of the Rab9-positive vesicles increased up to 800 nm (Fig. 5d), like the average diameter of autophagosomes. These results suggest that TMEM9 promotes the generation of Rab9-dependent autophagic vacuoles.

To further investigate whether TMEM9 is involved in Rab9-dependent alternative autophagy, we examined the subcellular localization of TMEM9-GFP and RFP-Rab9 together with Beclin1 under basal and autophagy-inducing conditions. In contrast to LC3, TMEM9 largely colocalized with Rab9-positive vesicles and Beclin1 even under basal conditions and the areas of their colocalization gradually increased under autophagy-activating conditions, such as EBSS treatment (Fig. 5e and f). In contrast to WT TMEM9, glycosylation-defective TMEM9-3NQ mutant colocalizes with neither RFP-Rab9 nor Beclin1 under basal and autophagy-activating conditions (Fig. 5e and f). Taken together, these results support the conclusion that TMEM9 participates in Rab9-dependent alternative autophagy rather than LC3-dependent conventional autophagy.

## Discussion

**Fig. 6:**
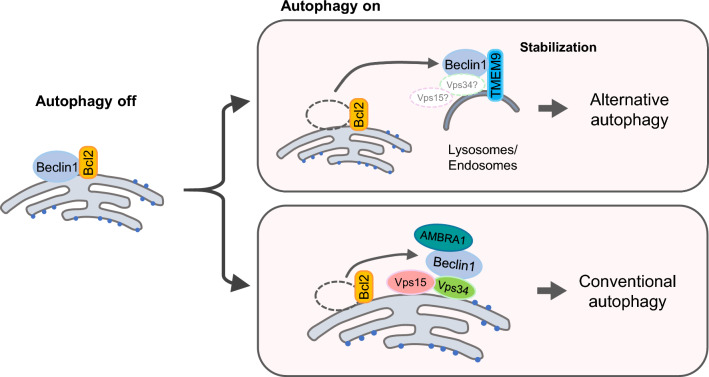
The proposed model shows the role of the TMEM9-Beclin axis in Rab9-dependent alternative autophagy. Under autophagy-inhibitory conditions, Beclin1 interacts with Bcl-2. Upon activation, TMEM9 on the endo/lysosomes replaces Bcl-2 in the Beclin1 complexes, leading to the activation of alternative autophagy utilizing Rab9

We demonstrate that TMEM9 physically interacts with Beclin1 through the cytosolic region of TMEM9 and the BH3 domain of Beclin1. This interaction triggers the dissociation of Bcl-2 from Beclin1, activating autophagy. Although the cytosolic region of TMEM9 directly interacts with Beclin1, the glycosylation of TMEM9 in the lysosomal luminal region is also essential for its lysosomal localization and interaction with Beclin1. TMEM9 plays a role in generating Rab9-dependent alternative autophagosomes rather than LC3-dependent autophagosomes. Our results suggest that the TMEM9-Beclin1 complex is the first identified alternative autophagy regulator within the diverse Beclin1 interactomes (Fig. 6).

Beclin1 acts like a scaffolding protein, associating with phosphatidylinositol-3-kinase to regulate the binding of its diverse partners. This interaction is crucial for constituting the nucleation core complex and for the kinase to generate Ptdlns3P, initiating autophagosome generation [27]. Depending on interacting partners, the Beclin1 complex determines diverse cellular fates by regulating autophagy in response to various external stimuli. Although Beclin1 is a common upstream regulator in both conventional and alternative autophagy, the role of Beclin1 in the regulation of alternative autophagy is not well understood [6]. In regulating alternative autophagy, TMEM9 may alter vesicle properties from endosomes to autophagic vacuoles. Specifically, the diameter of Rab9-positive vesicles is enlarged by TMEM9 expression (Fig. 5b–d). At this moment, we do not know how TMEM9 on the endo/lysosomes modulates autophagosome together with Beclin1. Considering the role of PI3K in autophagy and vesicle trafficking [28, 29], further studies on the TMEM9-dependent subcellular location and function are needed to delineate alternative autophagy.

This study highlights the importance of TMEM9 in Rab9-dependent autophagy through showing the colocalization of TMEM9 with Rab9. This is intriguing because a significant portion of LC3 generally colocalizes with lysosomes during the fusion process between autophagosomes and lysosomes [30]. GFP-LC3 does not colocalize with TMEM9. Instead, GFP-LC3 is observed in close proximity to TMEM9. Currently, we do not know why these two proteins are observed in close proximity rather than overlapping. In this Rab9-dependent alternative autophagy, it will be interesting to elucidate whether TMEM9-positive lysosomes differ from general lysosomes.

The function of TMEM9 has been primarily reported only in tumorigenesis. TMEM9 controls lysosomal acidification through its interaction with v-ATPase, promoting certain target proteins to be degraded, such as APC in Wnt/β-catenin signaling, thereby triggering tumorigenesis [14]. The v-ATPase plays a role in the acidification of endosomes as well as lysosomes and is associated with endosome trafficking [31]. It will also be interesting to identify a mode of the molecular interactions between Beclin1 and TMEM9 along with v-ATPase in regulating alternative autophagy. Besides lysosomal acidification in tumorigenesis, the role of TMEM9 is still largely unknown. Thus, the identification of another role of TMEM9 in the Rab9-dependent alternative autophagy opens new directions in a variety of signaling, including tumorigenesis. Although the detailed mechanism through which TMEM9 activates alternative autophagy is not yet clear, the ability of TMEM9 to generate larger Rab9 vesicles is remarkable and provides a hint. When alternative autophagy is analyzed in other models, the size of Rab9 vesicles, as well as the number of Rab9 vesicles, would be important. It should be noted that although TMEM9 colocalizes with Rab9, these proteins do not physically interact with each other.

The role of autophagy in tumorigenesis is like a double-edged sword and controversial. In general, autophagy suppresses tumorigenesis by inhibiting cancer cell survival and inducing cell death, but it also promotes tumorigenesis by enhancing cancer cell proliferation and tumor growth, depending on the properties of the tumor and stages [32]. Genotoxic stress triggers the phosphorylation status of Ulk1 to the alternative autophagy activating condition [5]. Compared to autophagy, the role of alternative autophagy in tumorigenesis is largely unknown and requires massive investigations. Our observation that the tumor-promoting protein TMEM9 can regulate alternative autophagy provides new insight into the role of alternative autophagy in tumorigenesis.

In summary, we demonstrate that the interaction between TMEM9 and Beclin1 promotes Rab9-dependent alternative autophagy by dissociating Bcl-2 from Beclin1. Although TMEM9 mainly localizes in lysosomes, it colocalizes with Rab9, not with GFP-LC3, in both basal and autophagy-inducing conditions. We propose a novel connection between Beclin1 interactomes and Rab9-dependent alternative autophagy.

### Supplementary Information

Below is the link to the electronic supplementary material.

Supplementary Figure S1. TMEM9 largely colocalizes with LAMP1 both in basal and autophagy-inducing conditions. HeLa cells were co-transfected with TMEM9-RFP (a and b) or TMEM9-GFP (c) and either GFP-Rab5 (a), GFP-Rab7 (b), or LAMP1-mCherry (c) for 24 h, incubated with basal medium, EBSS medium, or 10 μM TAT-Beclin1 for 6 h, and observed under confocal microscope. Scale Bar, 5 μm. The % of colocalization between Green and Red dots was quantified (d). Supplementary Figure S2. Glycosylation is important for the lysosomal localization of TMEM9. (a) The protein structure of TMEM9 was predicted using Alphafold based on sequence information. (b) Schematic representation of TMEM9 point mutants. (c) HEK293T cells were transfected with TMEM9 WT and various mutant forms as indicated for 24 h and analyzed with western blotting using anti-Flag and GAPDH antibodies. (d) HEK293T cells were transfected with TMEM9 WT and glycosylation defect mutants as indicated for 24 h and then treated with Tunicamycin for 12 h. Cell lysates were analyzed with western blotting using anti-Flag and anti-GAPDH antibodies. (e) HEK293T cells were transfected with Beclin1-HA and either flag-tagged TMEM9 or its point mutants for 48 h. Cell lysates were immunoprecipitated (IP) using anti-flag beads and analyzed by western blotting using anti-flag and anti-HA antibodies. (f) HEK293T cells were transfected with Beclin1-HA and either flag-tagged TMEM9 or its point mutants for 48 h and incubated with basal medium or EBSS medium for 6 h. Cell lysates were immunoprecipitated using anti-flag beads and analyzed by western blotting using anti-flag and anti-HA antibodies. (g) HeLa cells were transfected with TMEM9-GFP or its point mutations and LAMP1-mCherry for 24 h and incubated with basal medium or EBSS medium for 6 h. The cells were observed under the confocal microscope. Scale bar, 5 μm. Supplementary Figure S3. TMEM9 does not induce LC3-dependent autophagy. (a and b) HeLa and HeLa/shTMEM9 stable cells were transfected with GFP-LC3 for 24 h and then observed under the fluorescent microscope (a). The numbers of LC3 dots per cell were counted (b) (PDF 2631 KB)

## Data Availability

All raw data that support the findings of this study are available from the corresponding authors on reasonable request.

## Abbreviations

Atg: Autophagy-related
BiFC: Biomolecular fluorescence complementation
PtdIns3K: Class III phosphatidylinositol 3-kinase
ER: Endoplasmic reticulum
PTMs: Post-translational modifications
v-ATPase: Vacuolar-ATPase
VPS: Vacuolar protein sorting-associated protein
