# Supplementary material for: TMEM9 activates Rab9-dependent alternative autophagy through interaction with Beclin1
Source: Cell Mol Life Sci. 2024 Jul 30;81(1):322. doi: 10.1007/s00018-024-05366-1 (PMC11335249; doi:10.1007/s00018-024-05366-1)

Supplementary Figure S1. TMEM9 largely colocalizes with LAMP1 both in basal and autophagy-inducing conditions

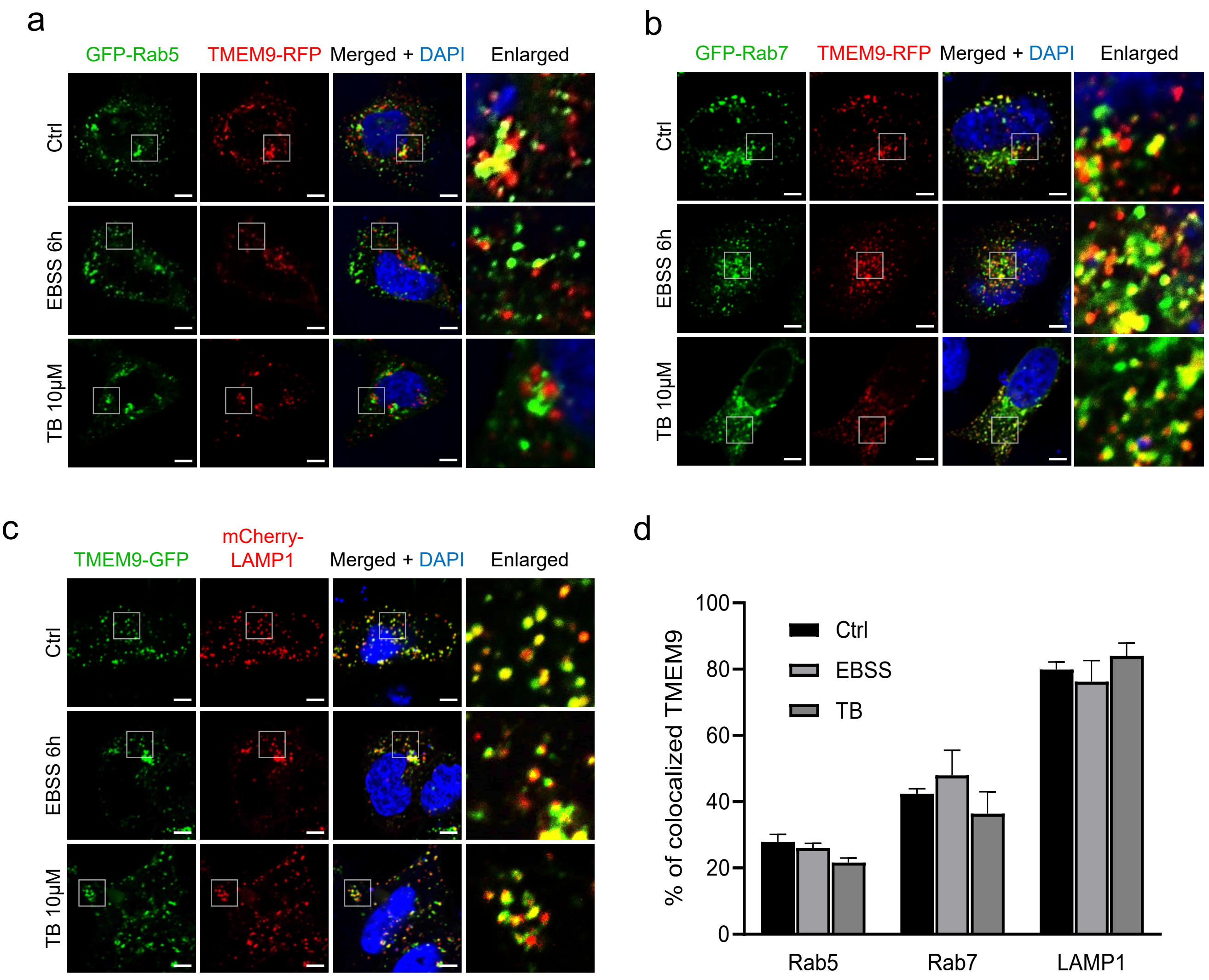

Supplementary Figure S2. Glycosylation is important for the lysosomal localization of TMEM9

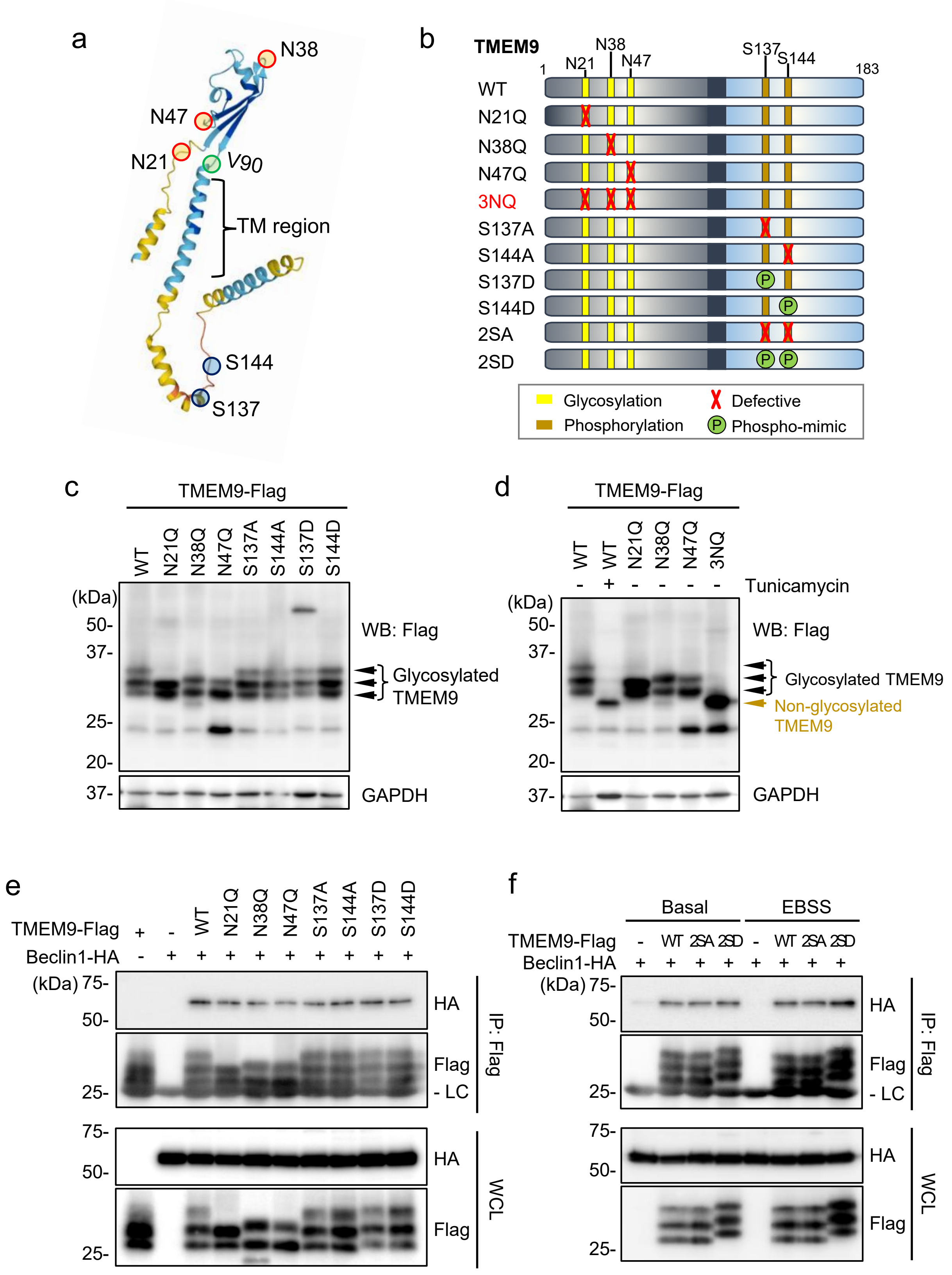

Supplementary Figure S2. Glycosylation is important for the lysosomal localization of TMEM9

g

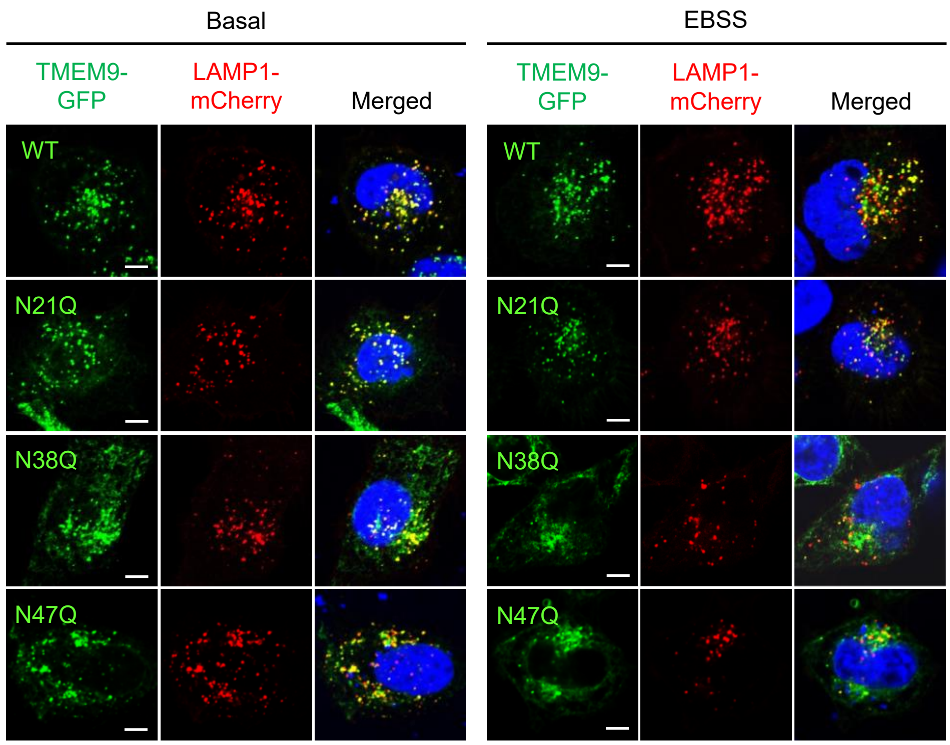

Supplementary Figure S3. TMEM9 does not induce LC3-dependent autophagy

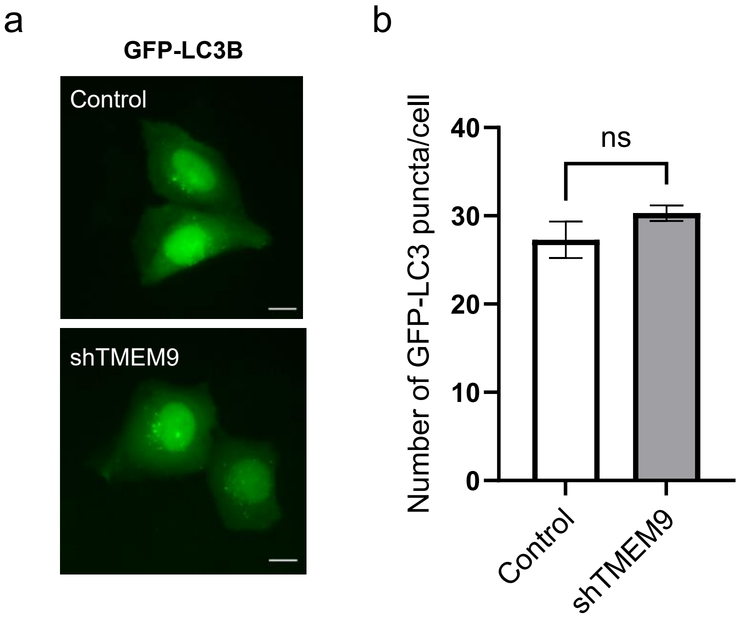

Supplement: Supplementary file 1 — Supplementary Figure S1. TMEM9 largely colocalizes with LAMP1 both in basal and autophagy-inducing conditions. HeLa cells were co-transfected with TMEM9-RFP (a and b) or TMEM9-GFP (c) and either GFP-Rab5 (a), GFP-Rab7 (b), or LAMP1-mCherry (c) for 24 h, incubated with basal medium, EBSS medium, or 10 μM TAT-Beclin1 for 6 h, and observed under confocal microscope. Scale Bar, 5 μm. The % of colocalization between Green and Red dots was quantified (d). Supplementary Figure S2. Glycosylation is important for the lysosomal localization of TMEM9. (a) The protein structure of TMEM9 was predicted using Alphafold based on sequence information. (b) Schematic representation of TMEM9 point mutants. (c) HEK293T cells were transfected with TMEM9 WT and various mutant forms as indicated for 24 h and analyzed with western blotting using anti-Flag and GAPDH antibodies. (d) HEK293T cells were transfected with TMEM9 WT and glycosylation defect mutants as indicated for 24 h and then treated with Tunicamycin for 12 h. Cell lysates were analyzed with western blotting using anti-Flag and anti-GAPDH antibodies. (e) HEK293T cells were transfected with Beclin1-HA and either flag-tagged TMEM9 or its point mutants for 48 h. Cell lysates were immunoprecipitated (IP) using anti-flag beads and analyzed by western blotting using anti-flag and anti-HA antibodies. (f) HEK293T cells were transfected with Beclin1-HA and either flag-tagged TMEM9 or its point mutants for 48 h and incubated with basal medium or EBSS medium for 6 h. Cell lysates were immunoprecipitated using anti-flag beads and analyzed by western blotting using anti-flag and anti-HA antibodies. (g) HeLa cells were transfected with TMEM9-GFP or its point mutations and LAMP1-mCherry for 24 h and incubated with basal medium or EBSS medium for 6 h. The cells were observed under the confocal microscope. Scale bar, 5 μm. Supplementary Figure S3. TMEM9 does not induce LC3-dependent autophagy. (a and b) HeLa and HeLa/shTMEM9 stable cells were tr [file 18_2024_5366_MOESM1_ESM.pdf]
